# Longitudinal oral and craniofacial follow-up of a genetically confirmed girl with Potocki-Lupski syndrome and repaired complete cleft palate: a case report

**DOI:** 10.3389/froh.2026.1918677

**Published:** 2026-09-10

**Authors:** Mariana C. Morales-Chavez, Ana M. Divasson-Pereira, Nancy Galindez-Landaeta

**Affiliations:** 1 Dental School, Dental Research Center, Santa Maria University, Caracas, Venezuela; 2 Dental School, Santa Maria University, Caracas, Venezuela

**Keywords:** cleft palate, craniofacial development, longitudinal follow-up, multidisciplinary care, pediatric dentistry, Potocki–Lupski syndrome

## Abstract

**Introduction:**

Longitudinal data describing the oral and craniofacial development of patients with Potocki–Lupski syndrome (PTLS) are scarce, particularly in individuals with repaired cleft palate. We report the long-term dental and craniofacial follow-up of a genetically confirmed Venezuelan girl with PTLS and repaired complete cleft palate, highlighting the contribution of continuous pediatric dental care within a multidisciplinary craniofacial team.

**Case report:**

A girl with repaired complete cleft palate was followed from the neonatal period through the mixed dentition stage, following genetic confirmation of PTLS by chromosomal microarray analysis. Comprehensive multidisciplinary evaluation documented developmental delay, generalized hypotonia, cardiovascular anomalies, gastrointestinal dysfunction, and food sensitizations. Dental management included fabrication of a neonatal palatal obturator, comprehensive preventive care, restorative treatment, orthopedic intervention, and full-mouth rehabilitation under intravenous conscious sedation. Longitudinal clinical and radiographic follow-up demonstrated satisfactory oral health, normal permanent tooth development, mild residual maxillary transverse constriction, a skeletal Class II relationship, and a hyperdivergent craniofacial growth pattern.

**Conclusion:**

This case illustrates the complexity of oral and craniofacial development in PTLS associated with repaired complete cleft palate and underscores the value of longitudinal pediatric dental follow-up in documenting growth and guiding clinical management. Continuous dental surveillance integrated within a multidisciplinary team facilitated timely preventive, restorative, and interceptive interventions while supporting long-term oral health and functional rehabilitation. Additional longitudinal studies are needed to define the oral phenotype of PTLS better and strengthen the evidence guiding multidisciplinary care.

## Introduction

Potocki-Lupski syndrome (PTLS), also known as 17p11.2 duplication syndrome, is a rare genomic disorder caused by a recurrent microduplication involving chromosome 17p11.2, most commonly encompassing the dosage-sensitive RAI1 gene. PTLS is characterized by a broad spectrum of clinical manifestations, including developmental delay, hypotonia, intellectual disability, speech impairment, feeding difficulties, behavioral abnormalities, autism spectrum traits, congenital malformations, and multisystem involvement. The estimated prevalence is approximately 1 in 25,000 individuals. However, the true frequency is likely underestimated because of phenotypic variability and limited access to molecular diagnosis, particularly in low- and middle-income countries (1–7).

Craniofacial abnormalities constitute an important component of the PTLS phenotype. Reported features include a triangular or elongated face, broad forehead, micrognathia, high-arched palate, smooth chin, down-slanting palpebral fissures, malocclusion, and variable craniofacial dysmorphism. Nevertheless, detailed descriptions of the oral phenotype remain scarce, and only a limited number of publications have specifically addressed dental findings and clinical management in affected individuals. Consequently, the natural history of craniofacial growth, occlusal development, and functional adaptation in children with PTLS remains poorly understood, highlighting the need for comprehensive clinical characterization of additional patients (1, 4, 5, 8, 9).

Although cleft palate is not considered a typical feature of Potocki–Lupski syndrome (PTLS), its occurrence may have important implications for feeding, speech, craniofacial growth, and occlusal development. In affected children, craniofacial morphology may reflect the combined influence of the underlying genetic disorder, the congenital palatal anomaly, surgical repair, functional adaptation, and neuromuscular development. Distinguishing the relative contribution of these factors is therefore challenging. It underscores the need for individualized longitudinal follow-up within a multidisciplinary cleft and craniofacial team to optimize oral, functional, and quality-of-life outcomes (10, 11).

Beyond its craniofacial manifestations, PTLS is a multisystem disorder that may involve gastrointestinal, immunological, and neurodevelopmental abnormalities. Growing interest in the gut–immune axis has highlighted the potential contribution of intestinal barrier function, microbial dysbiosis, and immune regulation to the clinical expression of complex genetic disorders. Although these mechanisms remain incompletely understood and no causal relationship has been established in PTLS, they provide a relevant biological context for interpreting multisystem phenotypes (12–15)..

Here, we report the longitudinal follow-up of a genetically confirmed Venezuelan girl with Potocki–Lupski syndrome (PTLS) and repaired complete cleft palate from the neonatal period through the mixed dentition stage. This case provides a detailed description of her oral, dental, and craniofacial phenotype, together with the preventive, restorative, and interceptive management strategies implemented throughout growth and development. To our knowledge, this is the first genetically confirmed Venezuelan case of PTLS with comprehensive longitudinal dental follow-up and one of the few reports describing the evolution of craniofacial and occlusal development in a patient with repaired cleft palate. By documenting the long-term clinical course, this report highlights the value of early diagnosis, continuous dental surveillance, and interdisciplinary care in optimizing oral health and functional outcomes in children with rare neurodevelopmental disorders.

## Case presentation

### Patient information

A 10-year-old girl with a history of complete cleft palate was referred during the neonatal period to a private dental clinic specializing in the comprehensive care of children with special health care needs. Following primary cleft palate repair, she remained under continuous interdisciplinary follow-up to monitor craniofacial growth, oral development, functional rehabilitation, and overall health.

### Medical history and genetic diagnosis

**Figure 1:**
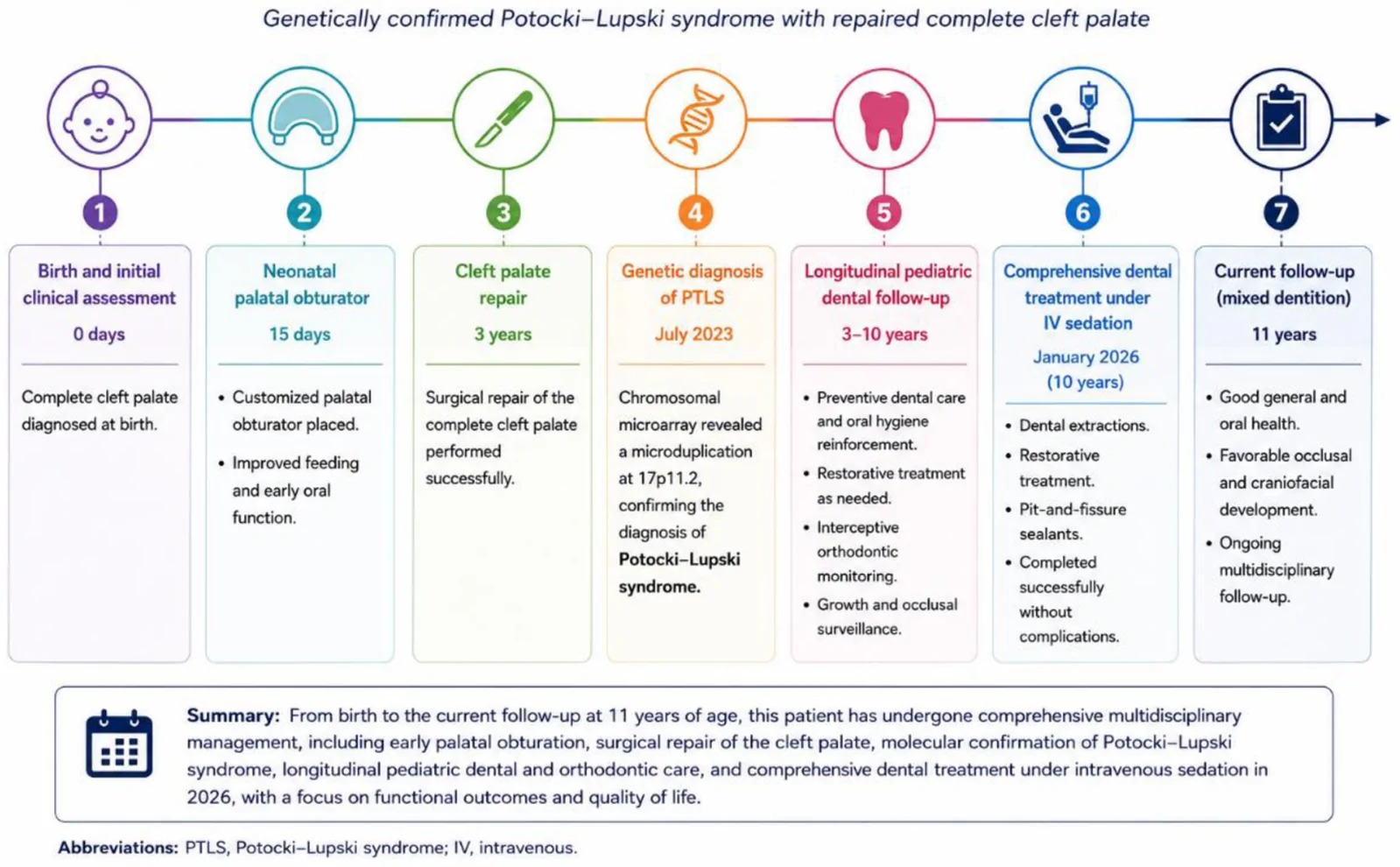
Longitudinal timeline of the patient's clinical and dental management from birth through the mixed dentition stage.

During early childhood, progressive developmental delay, generalized hypotonia, speech impairment, gastrointestinal dysfunction, and congenital cardiovascular abnormalities became evident, raising the suspicion of an underlying syndromic disorder. Comprehensive genetic evaluation performed in July 2023 by chromosomal microarray analysis identified a pathogenic duplication involving chromosome 17p11.2, confirming the diagnosis of Potocki-Lupski syndrome (PTLS) (Figure 1).

### Systemic assessment

Following the genetic diagnosis, the patient underwent coordinated evaluation by pediatric cardiology, gastroenterology, immunology, nutrition, pediatric surgery, speech therapy, anesthesiology, orthodontics, and pediatric dentistry.

Cardiologic assessment revealed a bicuspid aortic valve and mild mitral valve prolapse without hemodynamic compromise, requiring periodic surveillance. Persistent abdominal distension, chronic constipation, and feeding intolerance prompted comprehensive gastrointestinal investigation. Abdominal ultrasonography and plain abdominal radiography revealed marked fecal retention associated with diffuse gaseous colonic distension. Anorectal manometry confirmed preservation of the rectoanal inhibitory reflex, excluding Hirschsprung disease and supporting the diagnosis of functional constipation.

Immunological evaluation identified multiple food sensitizations, leading to individualized dietary modification, nutritional counseling, and allergen-specific immunotherapy. Overall, these findings illustrated the multisystem involvement characteristic of PTLS and emphasized the importance of coordinated long-term multidisciplinary care.

### Therapeutic intervention

Management began during the neonatal period with fabrication of a palatal obturator to facilitate feeding before primary cleft palate repair. Following surgical correction, the patient was enrolled in a comprehensive preventive oral health program consisting of individualized oral hygiene instruction, topical fluoride applications, dietary counseling, and periodic clinical evaluations.

At approximately 4 years of age, orthopedic treatment was initiated to support maxillary growth and guide occlusal development during craniofacial maturation. Because of the complexity of the required dental treatment and the patient's medical and behavioral characteristics, comprehensive oral rehabilitation was subsequently completed under ambulatory intravenous conscious sedation without perioperative complications.

Throughout the follow-up period, dental treatment was closely coordinated with the patient's medical team, ensuring an integrated approach to the management of both oral and systemic manifestations of PTLS.

### Dental and craniofacial findings

At the most recent clinical evaluation, extraoral examination revealed satisfactory facial symmetry, a mildly convex facial profile, and mild mandibular retrusion.

**Figure 2:**
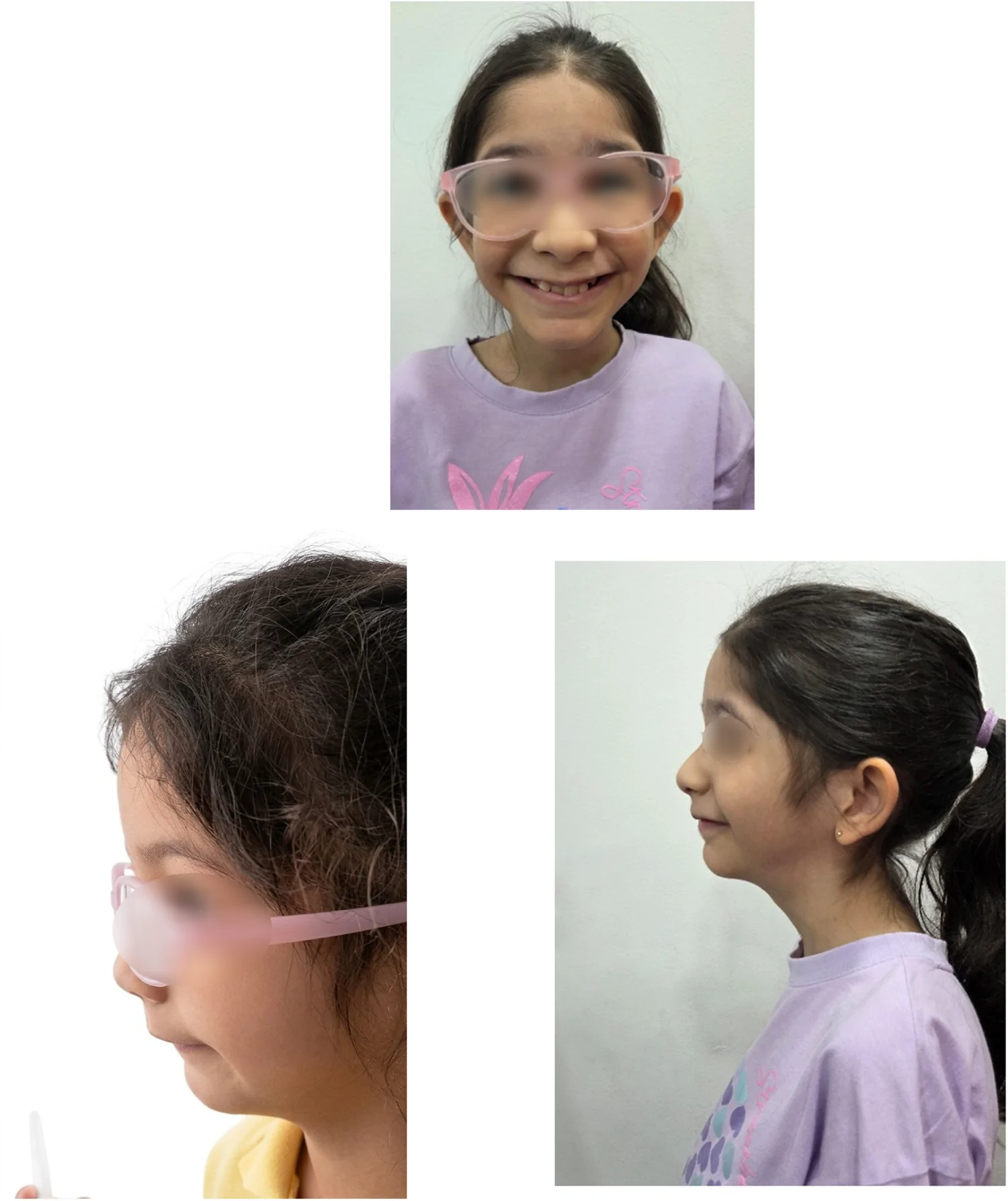
Clinical photographs. Extraoral photographs at ages 4 and 10.

**Figure 3:**
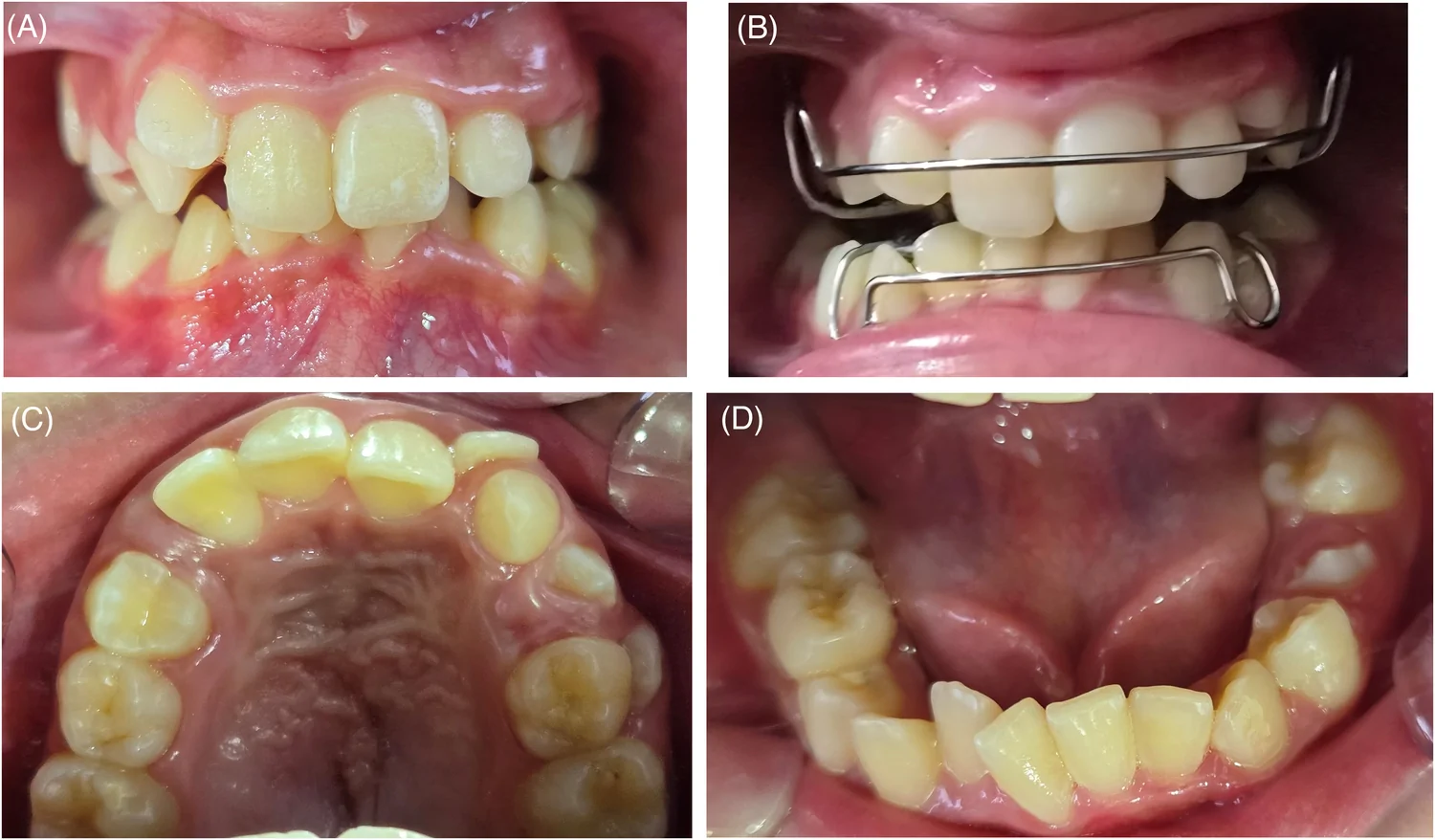
Intraoral clinical photographs obtained during the mixed dentition stage. **(A)** Frontal dental photograph; **(B)** frontal dental photograph with the orthodontic appliance; **(C)** and **(D)** lateral dental photographs.

Intraoral examination demonstrated mixed dentition with erupting permanent canines, mild residual anterior crowding, a well-healed repaired palate without clinically evident oronasal fistula, and mild residual transverse maxillary constriction. Oral hygiene was satisfactory, with no evidence of active dental caries or clinically significant periodontal inflammation (Figures 2 and 3).

### Radiographic assessment

**Figure 4:**
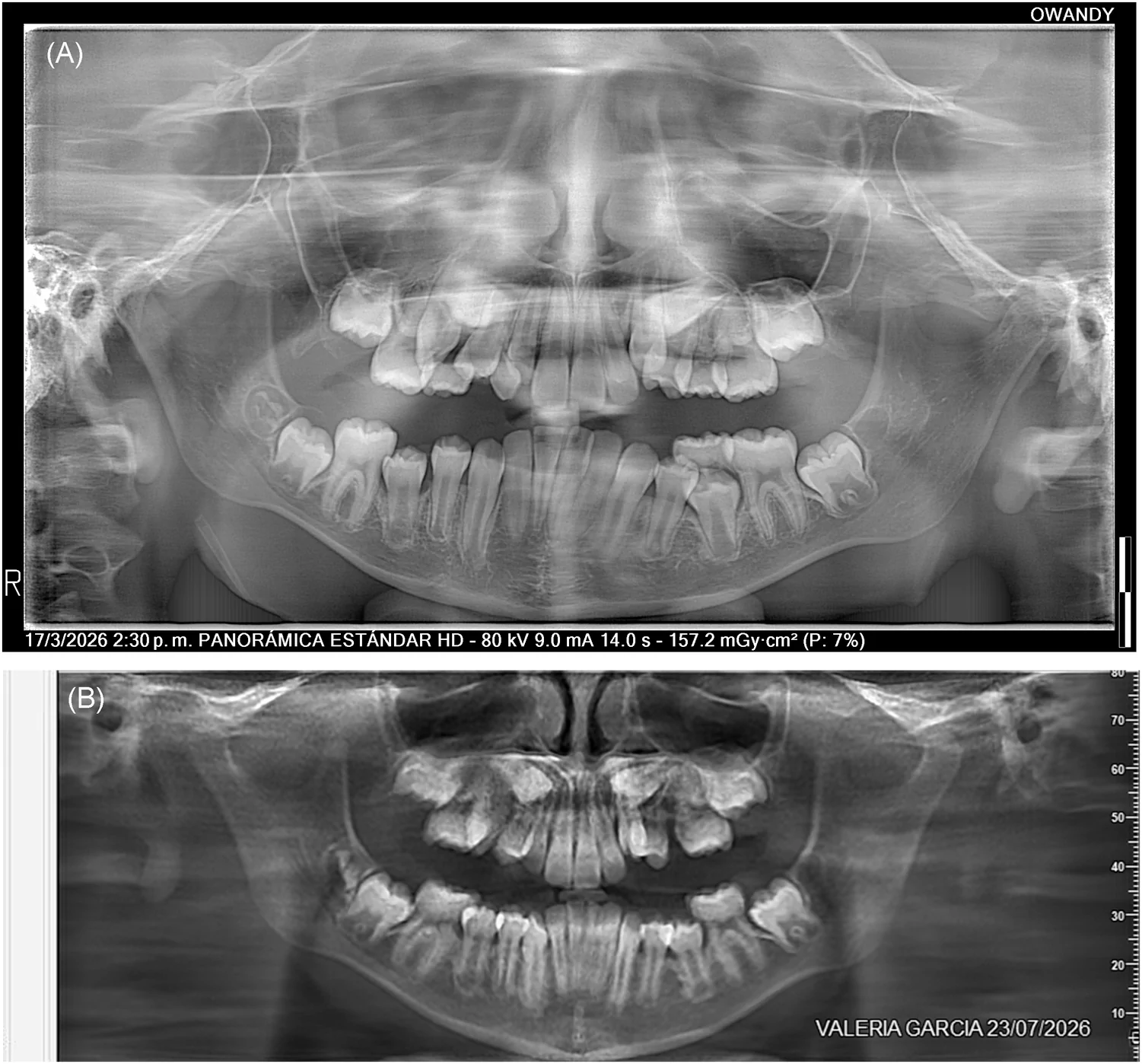
Radiographic evaluation during longitudinal follow-up. **(A)** Initial panoramic radiograph obtained before comprehensive dental treatment, demonstrating the mixed dentition stage and treatment needs. **(B)** Follow-up panoramic radiograph obtained six months after treatment, showing satisfactory healing following dental extractions, normal eruption of the permanent dentition, and the absence of postoperative complications.

Baseline panoramic radiography obtained during the early mixed dentition demonstrated normal development of the permanent dentition, without evidence of dental agenesis or other clinically significant developmental abnormalities. Follow-up panoramic imaging confirmed continued dental development consistent with the patient's chronological age (Figure 4).

**Figure 5:**
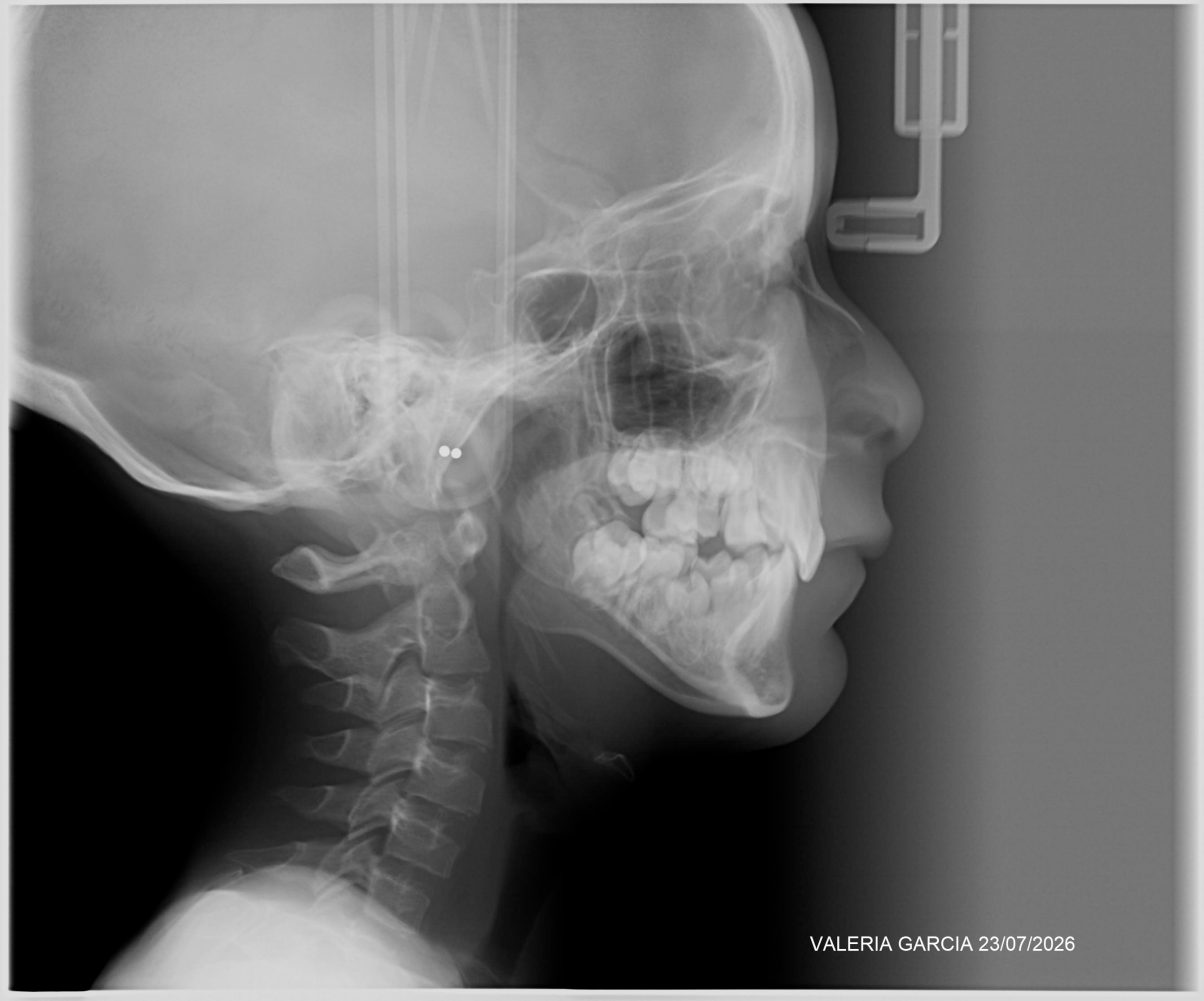
Lateral cephalometric radiograph demonstrating a skeletal class II relationship associated with mild mandibular retrusion and a hyperdivergent growth pattern.

Manual lateral cephalometric analysis revealed a skeletal Class II relationship characterized by a normally positioned maxilla (SNA = 84°), mild mandibular retrusion (SNB = 77°), and an increased sagittal skeletal discrepancy (ANB = 7°). Vertical skeletal assessment demonstrated a hyperdivergent growth pattern with an increased SN–mandibular plane angle (46°), whereas the SN–palatal plane angle (16°) remained within normal limits. These findings were consistent with the patient's facial morphology and clinical examination (Figure 5).

### Follow-up and outcomes

The patient continues to attend scheduled six-month recall visits for preventive dental care and monitoring of craniofacial growth and occlusal development. At the latest follow-up, oral health remained stable, with satisfactory oral hygiene, absence of active dental caries, preservation of the repaired palate, and age-appropriate dental development. Continued interdisciplinary follow-up has facilitated coordinated management of the oral and systemic manifestations of PTLS while supporting long-term functional stability and preventive oral health (Table 1).

**Table 1:** Summary of the main clinical findings and multidisciplinary management of the patient with Potocki-Lupski syndrome (PTLS).

Domain	Main findings
Genetic diagnosis	Potocki-Lupski syndrome confirmed by chromosomal microarray (17p11.2 duplication).
Craniofacial anomalies	Complete cleft palate surgically repaired during infancy; mild residual transverse maxillary constriction.
Neurodevelopment	Global developmental delay, generalized hypotonia, and speech impairment.
Cardiovascular	Bicuspid aortic valve and mild mitral valve prolapse without hemodynamic compromise.
Gastrointestinal	Functional constipation, abdominal distension, and feeding intolerance; Hirschsprung disease excluded by anorectal manometry.
Immunological	Multiple food sensitizations managed with dietary modification and allergen-specific immunotherapy.
Dental findings	Mixed dentition, erupting permanent canines, mild anterior crowding, satisfactory oral hygiene, and absence of active dental caries.
Radiographic findings	Normal permanent dentition development; skeletal Class II with mild mandibular retrusion and hyperdivergent growth pattern.
Long-term management	Multidisciplinary follow-up involving pediatric dentistry, orthodontics, genetics, cardiology, gastroenterology, immunology, nutrition, speech therapy, pediatric surgery, anesthesiology, and preventive oral care.

## Discussion

This report presents the longitudinal dental and craniofacial follow-up of a Venezuelan girl with genetically confirmed Potocki–Lupski syndrome (PTLS) and repaired complete cleft palate from birth through the mixed dentition stage. While individual oral manifestations of PTLS have been previously described, detailed longitudinal documentation of craniofacial growth, occlusal development, and pediatric dental management remains exceptionally limited. The present case provides a unique opportunity to examine how a rare genomic disorder, a congenital cleft palate, and its surgical repair interact throughout growth and development. Rather than suggesting a single etiologic explanation for the observed craniofacial phenotype, our findings emphasize the complex interplay between genetic background, surgical intervention, functional adaptation, and continuous dental care.

PTLS is characterized by marked clinical heterogeneity, with craniofacial manifestations varying considerably among affected individuals. Reported features include a triangular facial appearance, maxillary hypoplasia, high-arched palate, micrognathia, dental crowding, and malocclusion; however, detailed descriptions of dental and craniofacial development remain scarce. Complete cleft palate is an uncommon manifestation of PTLS, making the present case particularly relevant. Our patient demonstrated a combination of repaired complete cleft palate, skeletal Class II relationship, hyperdivergent facial pattern, mild maxillary transverse constriction, and dental crowding. Although similar findings have been reported individually in patients with PTLS or cleft palate, their coexistence in a single patient illustrates the phenotypic complexity of this rare syndrome. It reinforces the need for individualized longitudinal assessment (7, 10, 16).

Interpretation of the craniofacial findings requires caution because multiple biological and therapeutic factors likely contributed to the patient's growth pattern. Children with repaired cleft palate frequently exhibit alterations in maxillary growth related to both the congenital anomaly and surgical repair. In contrast, individuals with PTLS may also present hypotonia, developmental delay, altered oral motor function, and craniofacial dysmorphism. Consequently, it is not possible to determine the relative contribution of each factor to the skeletal Class II relationship, hyperdivergent pattern, or residual transverse maxillary constriction observed in this patient. Rather than supporting a single pathogenic mechanism, the present findings highlight the multifactorial nature of craniofacial development in children presenting with both PTLS and repaired cleft palate (8, 17, 18).

The most distinctive contribution of this report lies in its longitudinal follow-up from the neonatal period through the mixed dentition stage. Unlike most published reports of PTLS, which primarily describe clinical findings at a single point in time (8, 10, 16, 19), our case documents the patient's oral and craniofacial evolution from birth through the mixed dentition stage, providing insight into the dynamic interaction between growth, cleft palate repair, and comprehensive pediatric dental care. Early oral rehabilitation with a neonatal palatal obturator facilitated feeding before definitive palatal repair and was followed by continuous pediatric dental surveillance throughout childhood (20). This longitudinal approach enabled regular assessment of tooth eruption, oral hygiene, caries risk, occlusal development, and craniofacial growth, allowing preventive, restorative, and interceptive interventions to be implemented according to the patient's evolving clinical needs. As treatment requirements increased, comprehensive dental rehabilitation under intravenous sedation allowed definitive treatment to be completed safely while minimizing behavioral and psychological stress. Our experience supports the growing recognition that early and continuous pediatric dental involvement is an essential component of multidisciplinary care for children with syndromic craniofacial anomalies, contributing not only to oral health maintenance but also to functional rehabilitation and long-term treatment planning.

Another important aspect of this case is the multidisciplinary model of care that accompanied the patient throughout development. Management required close coordination among pediatric dentistry, orthodontics, craniofacial surgery, clinical genetics, pediatrics, speech therapy, and other healthcare professionals. Within this collaborative framework, pediatric dentistry extended beyond restorative treatment by providing continuous preventive care, monitoring craniofacial growth, educating caregivers, and contributing to long-term treatment planning. These observations support current recommendations advocating multidisciplinary, patient-centered care as the standard approach for children with cleft palate and other complex craniofacial conditions, particularly when associated with rare genetic disorders such as Potocki-Lupski syndrome (10, 21, 22).

Besides the craniofacial phenotype, our patient also exhibited gastrointestinal manifestations that required specialized evaluation during follow-up. These findings further illustrate the multisystem nature of PTLS (10); however, their relationship with the craniofacial and dental phenotype remains uncertain. Therefore, they should be interpreted as part of the overall clinical spectrum of the syndrome rather than as evidence of a causal biological mechanism. Future multidisciplinary studies integrating clinical, genetic, immunological, and molecular data may help clarify whether common biological pathways contribute to these apparently distinct manifestations (10, 13, 23).

This report should be interpreted in light of several limitations. As a single-case report, the present study cannot establish causal relationships between PTLS and the observed craniofacial characteristics. Furthermore, the simultaneous presence of repaired complete cleft palate makes it impossible to separate the individual effects of the genetic syndrome, congenital malformation, surgical repair, functional adaptation, and normal craniofacial growth. However, the extended duration of longitudinal data provides valuable information that is rarely available for patients with PTLS, offering unique insights into the oral and craniofacial development of affected individuals. This perspective remains underrepresented in the current literature (10, 16).

Despite these limitations, this report expands the limited literature describing the oral phenotype and craniofacial evolution of individuals with PTLS. More importantly, it underscores the value of continuous pediatric dental care integrated within a multidisciplinary craniofacial team from infancy through childhood. Longitudinal clinical follow-up not only supports timely preventive, restorative, and interceptive interventions but also contributes to a better understanding of craniofacial growth and oral development in this rare syndrome. Future longitudinal studies involving larger cohorts are needed to characterize the craniofacial spectrum of PTLS further and to optimize evidence-based preventive and therapeutic strategies for affected individuals (10, 16, 21).

## Conclusion

This case highlights the complexity of oral and craniofacial development in a child with genetically confirmed Potocki–Lupski syndrome and repaired complete cleft palate. Longitudinal follow-up from infancy through the mixed dentition provided valuable clinical insights into dental development, craniofacial growth, and the challenges associated with long-term management. The findings emphasize the importance of continuous pediatric dental care integrated within a multidisciplinary craniofacial team to support prevention, early intervention, and coordinated treatment planning. Additional longitudinal studies are needed to characterize the oral phenotype of PTLS better and to strengthen the evidence guiding clinical management of affected individuals.

## Data Availability

The raw data supporting the conclusions of this article will be made available by the authors, without undue reservation.
